# Modulation of the Tissue Expression Pattern of Zebrafish CRP-Like Molecules Suggests a Relevant Antiviral Role in Fish Skin

**DOI:** 10.3390/biology10020078

**Published:** 2021-01-22

**Authors:** Melissa Bello-Perez, Mikolaj Adamek, Julio Coll, Antonio Figueras, Beatriz Novoa, Alberto Falco

**Affiliations:** 1 Institute of Research, Development, and Innovation in Healthcare Biotechnology in Elche (IDiBE), Miguel Hernández University (UMH), 03202 Elche, Spain; melissa.bello@alu.umh.es; 2 Fish Disease Research Unit, Institute for Parasitology, University of Veterinary Medicine, 30559 Hannover, Germany; mikolaj.adamek@tiho-hannover.de; 3 Department of Biotechnology, National Agricultural and Food Research and Technology Institute (INIA), 28040 Madrid, Spain; juliocoll@inia.es; 4 Institute of Marine Research, Consejo Superior de Investigaciones Científicas (IIM-CSIC), 36208 Vigo, Spain; antoniofigueras@iim.csic.es (A.F.); beatriznovoa@iim.csic.es (B.N.)

**Keywords:** short pentraxins, c-reactive protein, zebrafish, transcript expression, antiviral, SVCV, rag1 mutants, skin, mucosal immunity

## Abstract

**Simple Summary:**

The clinical use of the human short pentraxin C-reactive protein as a health biomarker is expanded worldwide. The acute increase of the serum levels of short pentraxins in response to bacterial infections is evolutionarily conserved, as are the main functions of pentraxins. Interestingly, fish orthologs have been found to increase similarly after bacterial and viral stimuli, thus becoming promising candidates for health biomarkers of both types of infection in this group of vertebrates. To preliminarily assess their adequacy for this application, zebrafish and a fish rhabdovirus were chosen as infection model systems for the analysis of the levels of gene expression of all short pentraxins in healthy and infected animals in a wide range of tissues. Because some significant increases were found in skin (a very suitable sampling source for testing purposes), further transcript analyses were carried out in this tissue. Due to the functional similarities between pentraxins and antibodies, it was also checked whether short pentraxins can compensate for the deficiencies in adaptive immunity by using mutant zebrafish lacking this system. In conclusion, the obtained results suggest that short pentraxins are highly reactant against viruses in skin and their overexpression seems to reflect a mechanism to compensate for the loss of adaptive immunity.

**Abstract:**

Recent studies suggest that short pentraxins in fish might serve as biomarkers for not only bacterial infections, as in higher vertebrates including humans, but also for viral ones. These fish orthologs of mammalian short pentraxins are currently attracting interest because of their newly discovered antiviral activity. In the present work, the modulation of the gene expression of all zebrafish short pentraxins (CRP-like proteins, CRP1-7) was extensively analyzed by quantitative polymerase chain reaction. Initially, the tissue distribution of *crp1*-*7* transcripts and how the transcripts varied in response to a bath infection with the spring viremia of carp virus, were determined. The expression of *crp1*-*7* was widely distributed and generally increased after infection (mostly at 5 days post infection), except for *crp1* (downregulated). Interestingly, several *crp* transcription levels significantly increased in skin. Further assays in mutant zebrafish of recombinant activation gene 1 (*rag1*) showed that all *crp*s (except for *crp2*, downregulated) were already constitutively highly expressed in skin from *rag1* knockouts and only increased moderately after viral infection. Similar results were obtained for most *mx* isoforms (a reporter gene of the interferon response), suggesting a general overcompensation of the innate immunity in the absence of the adaptive one.

## 1. Introduction

Circulating pentraxins are considered pattern-recognition molecules that contribute to innate immunity by mainly facilitating the clearance of damaged cells and bacterial pathogens [1,2,3,4]. The representative forms of these molecules show an annular pentameric structural symmetry in humans [5]. Their monomers are characterized by the presence of a C-terminal domain of approximately 200 amino acid residues, containing the so-called “pentraxin signature”, a conserved 8 amino acid residue sequence (HxCxS/TWxS) [2,6,7]. When an additional N-terminal region is present, they are termed fusion or long pentraxins, and their prototype is pentraxin 3 (PTX3) [8]. In contrast, those soluble pentraxins consisting of just the C-terminal domain are termed classical or short pentraxins, and they include C-reactive protein (CRP) and serum-amyloid P component (SAP) [9].

The classification into CRPs and SAPs is mostly based on their differential ligand affinities in humans, although in other species this feature shows overlapping and even total reversion between the two groups [3,10,11,12,13,14,15]. Likewise, several oligomerization forms, other than pentameric, have also been identified across evolution, even in humans [16,17]. Nevertheless, because of shared homologous sequences corresponding to functionally important motifs and analogous molecular structures, fundamental activities associated with short pentraxins in humans are evolutionarily conserved [3,4,12,13,14,15]. One such activity is their role as reactive plasma proteins during the acute phase response (APR), an immediate and systemic physiological feedback of the innate immune system to trauma, injury, and infection [1,2,3,4]. In this regard, CRP is the predominant acute phase protein (APP) in most mammals and thus its clinical use as a health biomarker is common [1,2,3,4].

Although APRs are usually triggered during both bacterial and viral infections [18], increases of serum CRP levels in mammals are more characteristic of bacterial rather than viral infections [1,18,19]. In fish, short pentraxins are common ancestors of both mammalian CRP and SAP counterparts, which diverged very early in mammalian evolution after the separation of mammalian and avian lineages [20], and thus arose independently from a homologous differentiation in arthropods [7]. The existing studies analyzing the serum levels of fish short pentraxins (hereafter termed CRP-like proteins) show milder reactions than mammalian ones with similar response levels to bacterial and viral stimuli [21,22], which makes them potential biomarkers for both types of infection in fish. Interestingly, recent comparative studies of these ancestral orthologs have also revealed new interactions and activities such as affinity for different cholesterol metabolites, the modulation of autophagy, and antiviral activity [15,23,24,25].

In the present study, the transcript expression of all CRP-like protein (CRP1–7) encoding genes was analyzed extensively in zebrafish, which offers a great variety of scientific tools [26,27]. In addition to the determination of the tissue distribution of all *crp* transcripts and their modulation after infection with the spring viremia of carp virus (SVCV), they were also analyzed on the skin from zebrafish that are mutants of recombinant activation gene 1 (*rag1^+/+^* and *rag1^−/−^*). The *rag1^−/−^* mutants present a point mutation that generates a premature stop codon in the catalytic domain of this protein, at the endonuclease responsible for the V(D) J recombination [28]. This process results in functional macrophages, natural killer cells, and neutrophils and in a complete block of immunoglobulin gene assembly and T cell receptors (TCR) formation [29]. Moreover, it leads to the absence of adaptative immunity, providing a living platform to elucidate mechanisms of the innate immune responses [30]. Since CRPs have been previously referred to by other authors as ancient antibodies, because of their several analogous activities [2], this work aimed at investigating the behavior of these molecules in the absence of antibodies and at exploring their potential as health biomarkers of viral infections in fish.

## 2. Materials and Methods

### 2.1. Cell Lines and Virus

*Epithelioma papulosum cyprinid* (EPC) cells from fat-head minnow (*Pimephales promelas*) were purchased from the American Type Culture Collection (ATCC, Manassas, VI, USA, ref. no. CRL-2872). EPC cell monolayers were grown in Dutch-modified Roswell Park Memorial Institute (RPMI)-1640 culture medium (Sigma, St. Louis, MO, USA), supplemented with 10% fetal bovine serum (FBS) (Sigma), 2 mM glutamine, 1 mM sodium pyruvate, 50 μg/mL gentamicin, and 2 μg/mL of fungizone (Gibco BRL-Invitrogen, Carlsbad, CA, USA), at 28 °C in a 5% CO_2_ atmosphere.

SVCV isolate 56/70 from carp (*Cyprinus carpio*) was replicated in EPC cells at 22 °C without CO_2_ supply and by using previously described cell culture media except for 2% FBS (infection media). After 7 days post-infection (dpi), infective supernatants were harvested, clarified by centrifugation at 4 kg and 4 °C for 30 min, aliquoted, and stored at −80 °C until use. Virus titers in plaque forming units (pfu) were determined by the focus forming assay as described elsewhere [31].

### 2.2. Animals

The adult wild-type zebrafish (*Danio rerio*) were obtained by natural spawning from mating adults at one of the host institutions (Instituto de Investigaciones Marinas-CSIC, Vigo, Spain). Likewise, adult zebrafish that are mutants of recombinant activation gene 1 (*rag1^+/+^* and *rag1^−/−^*) were obtained at the University of Murcia (Murcia, Spain), and genotyped when they reached ~1 g (~6 months of age). Fish were maintained at 28 °C in 30-L aquaria, following established protocols [26], and fed daily with commercial food (Vipan BioVip, Berlin, Germany). Before the infection experiments, fish were acclimatized to 22 °C for 2 weeks. Prior to methodological handling, fish were anaesthetized by immersion in 100 mg/L tricaine methanesulfonate (MS-222) (Sigma). End-point fish euthanasia was performed by overdosing with tricaine methanesulfonate (500 mg/L).

### 2.3. Ethical Statement of Zebrafish Handling

All experiments with zebrafish complied with the Spanish Law for Animal Experimentation (Royal Decree-Law, 53/2013) and the European Union Council Directive 2010/63/UE. Animal trial procedures were approved by the local government ethics committee on animal experimentation (Dirección General de Agricultura, Ganadería y Pesca, Generalitat Valenciana), the Project Evaluation Board of Miguel Hernández University (permit no. UMH.IBM.JFG.01.14), and the CSIC National Committee on Bioethics (permit no. ES360570202001/16/FUN01/PAT.05/tipoE/BNG).

### 2.4. In Vivo Viral Infection

For each infection experiment, zebrafish were exposed to 10^4^ ffu/mL of SVCV by bath immersion for 90 min at 22 °C (optimal temperature for SVCV replication). Mock-infected zebrafish were incubated with equivalent volumes of cell culture medium in parallel experiments. Fish were then transferred to tanks with clean water and kept at 22 °C to allow for the progress of SVCV infection. Tissues were harvested at specific time points after infection for each experimental design. All tissues were dissected under a binocular loupe for transcript expression analysis.

For a general analysis of the tissue-specific transcription levels (tissue distribution assays) and their modulation in response to SVCV infection, four wild-type zebrafish were used for each condition; that is, four for the mock-infection, four for the 2-day infection, and four for the 5-day infection. Tissue samples from the mock-infected fish were collected at 2 dpi and used to determine the basal (and reference) expression. The tissues collected comprised head kidney (HK), liver, skin, gills, gut, muscle, and spleen.

To analyze the modulation of the transcription levels in skin from *rag1^+/+^* and *rag1^−/−^* zebrafish in response to SVCV, four individuals from each genotype were mock- and SVCV-infected. Skin samples from these fish were collected at 2 dpi to measure the early immune responses evoked by the virus entry and replication in one of its initial target tissues.

### 2.5. RNA Isolation, cDNA Synthesis, and qPCR

Total RNA was extracted by using EZNA HP Tissue RNA kits (Omega Bio-tek, Norcross, GA, USA) and subsequently treating the samples with DNase (Turbo DNA-free™ Kit, Ambion Inc., Austin, TX, USA) by following the manufacturer’s instructions. RNA concentrations were estimated with a Nanodrop 1000 spectrophotometer (Thermo-fisher Scientific, Waltham, MA, USA). Isolated RNA samples were stored at −80 °C until use.

For the synthesis of cDNA, 0.5 μg of isolated RNA from each sample and the Moloney murine leukemia virus (M-MuLV) reverse transcriptase were used (Gibco BRL-Invitrogen), as previously described elsewhere [22].

qPCR was performed by using an ABI PRISM 7300 thermocycler (Applied Biosystems, Branchburg, NJ, USA). Reactions were conducted in 20-μL-volume reactions comprising: 2 μL of cDNA, 900 nM of each corresponding forward and reverse primer (Sigma) (primer sequences are shown in Table S1), and 10 μL of SYBR Green PCR master mix (Life Technologies, Paisley, UK). Non-template controls were added for each gene analysis. All reactions were performed using technical duplicates. Cycling conditions were an initial denaturing step (10 min at 95 °C), followed by 40 cycles comprising 1 min at 65 °C and 1 min at 95 °C, and finally an extension step of 10 min at 65 °C. Melting curves were checked for inconstancies in each reaction. Cell and viral gene expression results were obtained and represented as relative transcription levels, by normalizing the expression level of each target gene to endogenous elongation factor 1-α (*ef1a*) levels by using a variation of Livak and Schmittgen’s method [32] by the formula 2^Ct ref.−Ct target^. For the analysis of the transcriptional responses to SVCV infection, the data are represented as fold changes relative to the corresponding samples from mock-infected individuals (*ef1a*-normalized expression in samples from infected individuals/*ef1a*-normalized expression in samples from mock-infected individuals), and the data lower than 1-fold were inverted and represented as negative values to show downregulation.

### 2.6. Statistical Analysis and Graphics

Data are shown as mean and standard deviation (SD). Resulting datasets were subjected to the most appropriate statistical analysis depending on each particular experimental design. Significant differences were determined by one-way ANOVA and Tukey’s multiple comparison (datasets in Section 3.1) or two-way ANOVA and Sidak’s multiple comparison test (datasets in Section 3.2 and Section 3.3). Prism v7 (Graphpad software, La Jolla, CA, USA) was used for creating the graphs and statistical analysis. *p* < 0.05, *p* < 0.01, and *p* < 0.001 statistical differences with respect to control groups are indicated in each graph by the letters a, b, and c, respectively. Further details are specified accordingly in the figure captions.

## 3. Results and Discussion

### 3.1. Broad Tissue Distribution of Zebrafish crp1–7 Expression

**Figure 1:**
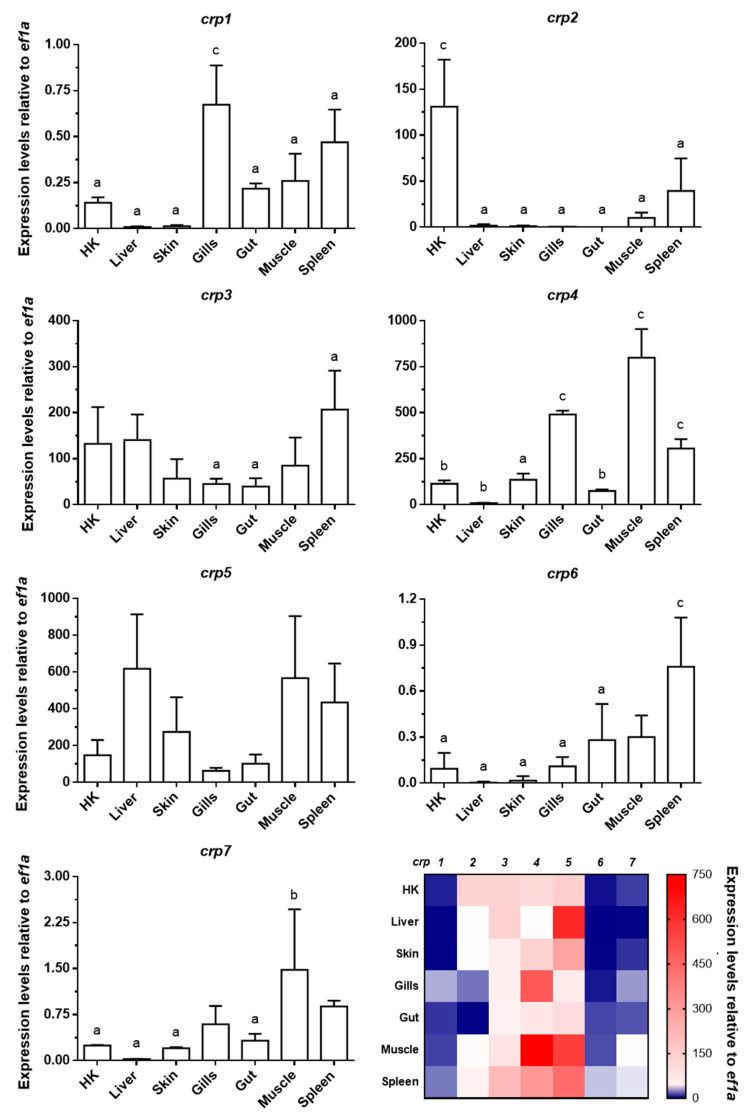
**Gene expression analysis of *crp1–7* in tissues of healthy zebrafish.** The expression of *crp1*–*7* was determined by RT-qPCR by using specific primers for each isoform (Table S1). *ef1a* mRNA was used as the endogenous control to normalize data, which are represented as the mean relative expression level × 10^3^ ± SD of four different individuals. Statistical differences (*p* < 0.05, one-way ANOVA) between tissues are represented by: a (different from up to 1–2 tissues), b (different from up to 3–4 tissues), and c (different from up to 5–6 tissues). Data in bar graphs are summarized in a final double gradient colormap (descending blue gradient for values from 0 to 1 and ascending red gradient from values from 1 to ≥750).

All seven *crp* isoforms were expressed in all the tested tissues (HK, liver, skin, gills, gut, muscle, and spleen) from healthy adult zebrafish, albeit with quite different expression patterns (Figure 1). Among all the isoforms, *crp3* and *crp5* showed the most consistent and broad expression patterns, since the site of expression significantly affected the transcription levels reached by the others (*p* < 0.05).

According to our results, the most pronounced expression level was found for *crp4* in muscle (799.95 ± 155.24), and the lowest one for *crp1* in liver (0.009 ± 0.003). The spleen and muscle tissues appeared to be among the major basal expression sites for all *crp*s. In general terms, our results confirm the broad extrahepatic expression of zebrafish *crp*s, in line with previous observations in wild-type zebrafish [25], as well as in other fish species such as *Salmo salar* [33], *Carassius auratus* [34], *Cyprinus carpio* [35], *Oplegnathus fasciatus* [36], *Plecoglossus altivelis* [37], *Sebastes schlegelii* [38], and *Cynoglossus semilaevis* [39,40]. Interestingly, the skin also showed notable expression levels for some isoforms, particularly *crp3–5*. A few studies previously reported the expression of *crp*s in skin from zebrafish [25], *Cyprinus carpio* [35,41], *Oplegnathus fasciatus* [36], and *Sebastes schlegelii* [38] and its presence in skin mucus from *Gadus morhua* [42], *Raja kenojei* [43], *Cyclopterus lumpus* [44], and *Tilapia mossambica* [45].

### 3.2. Systemic Modulation of Zebrafish crp1–7 Expression Levels in Response to SVCV Infection

**Figure 2:**
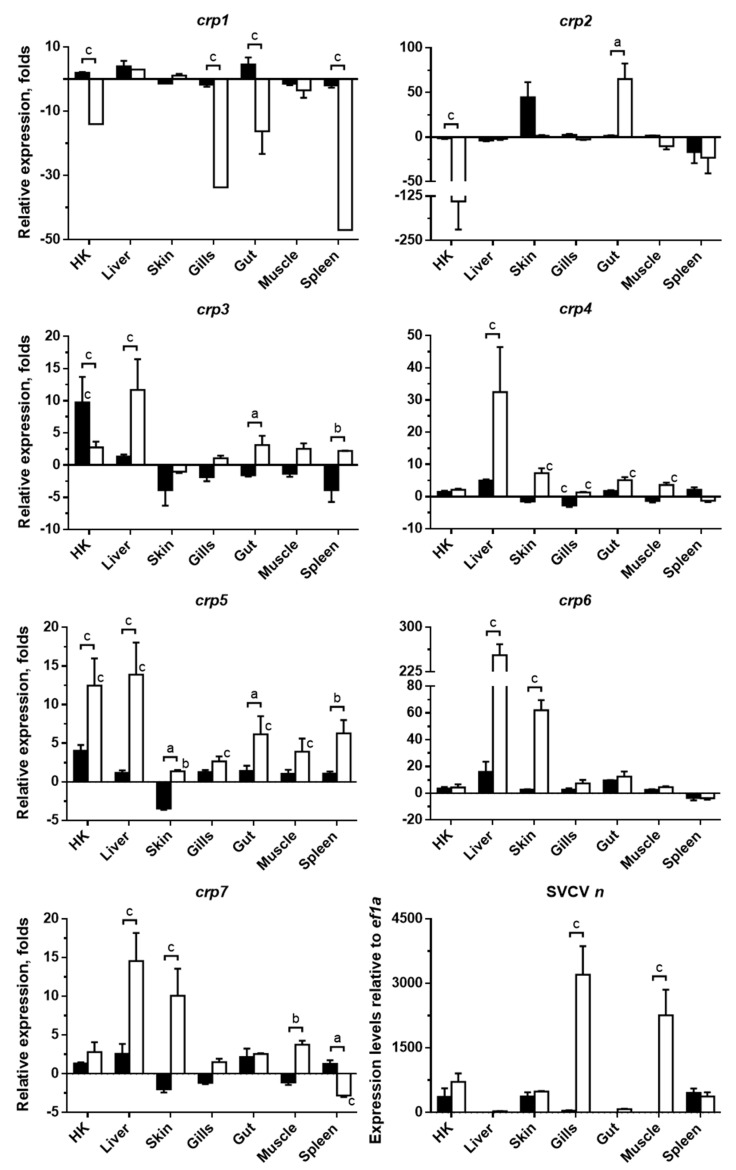
**Expression modulation of *crp1*–*7* in zebrafish tissues in response to spring viremia of carp virus (SVCV) infection.** The transcription levels of *crp1*–*7* and SVCV *n* in tissues from SVCV-infected zebrafish at 2 and 5 dpi (black and white bars, respectively) were quantified by RT-qPCR. *ef1a* mRNA was used as the endogenous control in all cases. *crp1*–*7* transcription levels were also normalized to the values obtained from the corresponding samples in non-infected fish. Data are represented as the mean fold changes ± SD for *crp*s and as the mean relative expression level ± SD for SVCV *n* (four different individuals in all cases). Significant differences were determined by two-way ANOVA and Sidak’s multiple comparison test. Statistical differences between the 2- and 5-dpi groups are represented by keys together with a, b, and c letters on top. Statistical differences between the 2- or 5-dpi groups and the non-infected group are represented by a, b, and c letters just on top of the corresponding bars. a, *p* ≤ 0.05; b, *p* ≤ 0.01; c, *p* ≤ 0.001.

The modulation of the *crp1*–*7* expression profiles at each tissue in response to SVCV was also analyzed at 2 and 5 dpi by qPCR (Figure 2), and it revealed that both the infection stage and the tissue tested significantly altered the expression levels of each *crp* (*p* < 0.001). As can be observed in Figure 2, SVCV infection resulted mostly in the upregulation of all *crp* isoforms, except for *crp1*, which was mostly downregulated. The highest increases occurred at 5 dpi, mainly for *crp6* in liver (253 ± 18 folds), *crp2* in gut (65 ± 18 folds), and *crp7* in skin (62 ± 8 folds), matching with the highest viral loads, as observed from the expression pattern of the SVCV *n* gene (Figure 2), which was similar to previously reported levels [46,47]. The SVCV infection levels at 2 dpi (Figure 2) were high in HK, spleen (the two most relevant lymphoid organs in fish [48]), and skin. In contrast, the lowest levels of SVCV *n* expression were found in the liver at both time points. The modulation of the expression of certain *crp* genes in response to viral infections has been previously described, in some particular tissues, for zebrafish infected with SVCV [49] and viral hemorrhagic septicemia virus (VHSV) [15,25], for *Cyprinus carpio* infected with cyprinid herpesvirus 3 (CyHV-3) [50] and injected with polyinosinic:polycytidylic acid (poly(I:C), which mimics viral infections) [22] and for *Cynoglossus semilaevis* [40] and *Oplegnathus fasciatus* [36] infected with red seabream iridovirus (RSIV), displaying a similar overall trend. However, the skin was not analyzed in any of those studies, and only one studied the gut, reporting increased levels of a carp *crp* isoform in this tissue in response to poly(I:C) [22].

In parallel, apart from the eventual but remarkable decrease in *crp2* transcripts in HK (−140 ± 79 folds), our results also revealed that SVCV infection downregulated the expression of *crp1* in HK, gills, gut, and spleen (the lowest, −47-fold) at 5 dpi, which could be associated with the fact that CRP1 in zebrafish is the only exclusively intracellular isoform, since it lacks the signal peptide. The low participation of CRP1 in not only viral responses but also viral neutralization has been previoulsy described, suggesting that CRPs should be secreted to be efficient against viral infections [25].

The obtained results on the expression of *crp*s also suggest that the liver is the major CRP-producing tissue in zebrafish in response to infection (Figure 2), as also occurs in mammals for CRP/SAP and other APPs when the APR is triggered [1,18,51,52]. Under this situation, the expression of CRP in mammals is principally induced in hepatocytes by the action of the cytokine interleukin-6 (IL-6), whose effect is boosted by interleukin-1 (IL-1) [18,51,52,53]. A similar mechanism might be occurring in fish, as *crp4* and *crp5* are induced in zebrafish embryos microinjected with the *il6* transgene [25] as is *crp1a* by recombinant cytokines *il1b* in primary HK cells from *Salmo salar* [33]. Furthermore, IL-6 has been found to increase in response to viral infections in both mammals [54,55,56] and fish [57,58]. In this vein, another parallelism to mammals is the observation of a wide extrahepatic expression of CRPs [18,59]. However, this event appears to be more relevant in fish as is inferred from the high basal (Figure 1) and SVCV-induced (Figure 2) levels of *crp* in the HK, gut, and, surprisingly, skin.

### 3.3. CRPs Are Constitutively Overexpressed in Skin in the Absence of Adaptive Immunity

The modulation of the expression of some zebrafish *crp*s in response to SVCV in skin, which had not been described before, attracted our attention. On the one hand, the skin is not considered a major lymphoid [48,60] nor APR-related [18,51] tissue. On the other hand, the skin is one of the preferential entry sites of rhabdovirus in fish [61,62], which explains its early and notable response. In addition, the skin is the largest immunologically active organ in fish and is a type of secondary lymphoid tissue called diffuse mucosa-associated lymphoid tissue (MALT), which is not only endowed with strong innate immune activity, but also with relevant adaptive immune properties [60,63,64,65].

In order to further characterize this effect in the skin of zebrafish and to explore a potential involvement of the adaptive immune system, a comparative study of the expression changes of *crp*s in skin in response to SVCV infection was performed in *rag1* mutants. In this experiment we analyzed the basal and SVCV-induced levels of *crp*s in *rag1^+/+^* and *rag1^−/−^* mutant zebrafish. At this point, it is worth mentioning that wild-type zebrafish (previous experiments) and the *rag1^+/+^* zebrafish (following experiments) may differ at some level in the expression of certain genes due to the fact that the former ones include not only homo, but also heterozygous *rag1* individuals (*rag1^+/+^* and *rag1^+/−^*), which may be affected by transcriptional compensatory mechanisms as they are partially deficient in the generation of a mature adaptive immune response [28].

**Figure 3:**
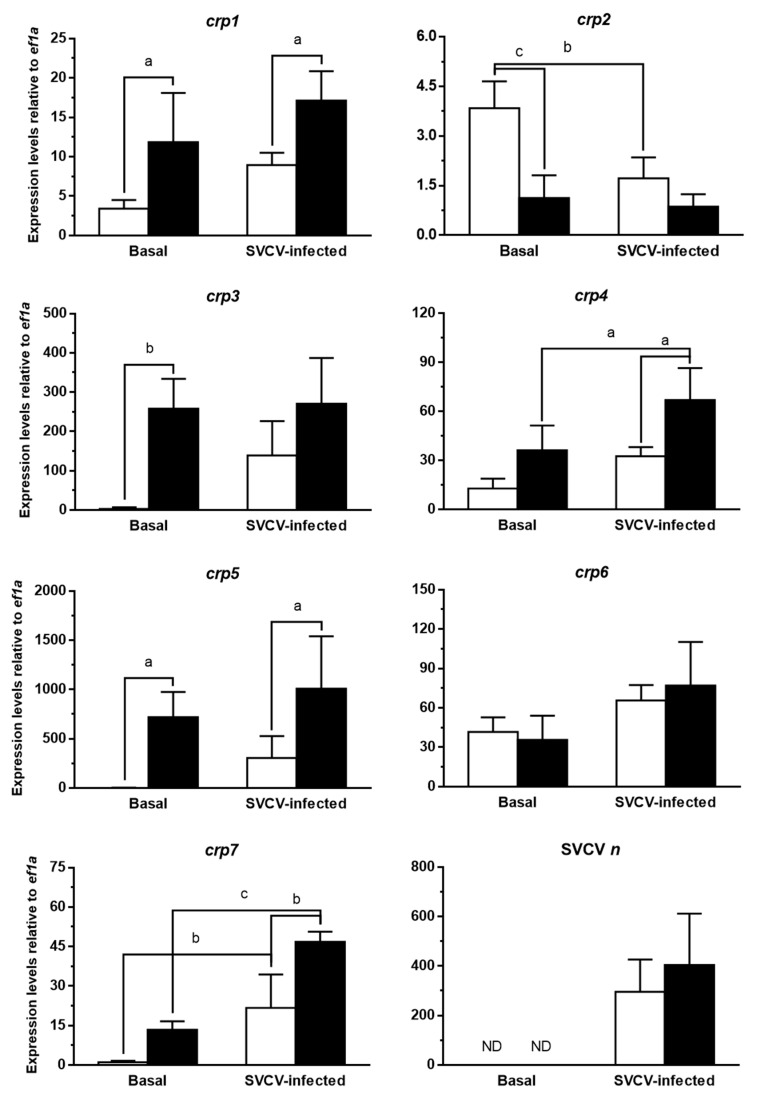
**Expression modulation of *crp1*–*7* in skin from *rag* mutant zebrafish in response to SVCV infection.** The transcription levels of *crp1*–*7* and SVCV *n* were quantified by RT-qPCR in the skin of *rag^+/+^* (white bars) and *rag^−/−^* (black bars) mutant zebrafish at 2 dpi with SVCV. *ef1a* mRNA was used as the endogenous control to normalize data, which are represented as the mean relative expression level (× 10^3^ for *crp*s) ± SD of four different individuals. Significant differences were determined by two-way ANOVAs and Sidak’s multiple comparison test. Statistical differences between the experimental groups are represented by keys together with a, b, and c letters on top. a, *p* ≤ 0.05; b, *p* ≤ 0.01; c, *p* ≤ 0.001; ND, not detected.

The results (Figure 3) show that basal expression levels of *rag1^−/−^* zebrafish were higher for *crp1, 3,* and *5*, and lower for just *crp2*, in comparison to that of *rag1^+/+^* zebrafish. After viral challenge, the expression levels of all *crp*s, except for *crp2* (which continued to be downregulated), appeared to increase in *rag1^+/+^* fish, although only the *crp7* transcription levels were found to be significantly upregulated in this experiment. Regarding *rag1^+/+^* and *rag1^−/−^* infected groups of zebrafish, expression differences between them were similar to those found at basal conditions, i.e., *crp1, 4*, *5,* and *7* expression levels were upregulated in *rag1^−/−^* zebrafish, while no differences were detected among their viral loads. When comparing the effect of the SVCV infection on the *rag1^−/−^* group, only the expression levels of *crp4* (from 36.25 to 66.90) and *crp7* (13.40 to 46.76) were modulated, suggesting that most *crp* basal expression in this group might be already close to maximum levels.

The modulation of the transcription levels of *mx*, an interferon (IFN) stimulated gene (ISG) often used as a marker of the activation of the type I IFN response, was also analyzed in these samples, resulting in similar results as those previously observed for *crp* genes (Supplementary Figure S1). Thus, the determination of the expression levels of *mxa*-*g* zebrafish isoforms confirmed that the IFN response to the viral infection was triggered in skin from experimental fish, and that the basal expression of most *mx* isoforms in *rag1^−/−^* fish had also reached similar levels to those found after SVCV infection. This observation is in agreement with previous evidence indicating that basal immunological status is elevated in *rag1^−/−^* mutants and extends to skin, as well as the parallel modulation of multigene families associated to the antiviral response [66]. In the case of *crp* genes, such compensatory effects might be due not only to their recently described antiviral activity [23,24,25,40] but also their evolutionary-conserved antibody-like activities [15,67,68,69]. In addition, the upregulation of this type of genes in *rag1^−/−^* mutants has been associated with epigenetic reprogramming [70], similar to the underlying mechanisms regulating innate trained immunity [71,72], and apparently also occurring in the *crp* genes in zebrafish [73].

## 4. Conclusions

The present study aimed at contributing to the current knowledge on fish short pentraxins by describing extensively the tissue distribution of the transcript expression of all seven zebrafish *crp* genes and their modulation in each tissue, especially skin, in response to infection with SVCV. Thus, all *crp*s were found to be constitutively expressed in all tested tissues (i.e., HK, liver, skin, gills, gut, muscle, and spleen). In general terms, *crp4* and *crp5* presented the highest levels of expression, and gills, muscle, and skin were the major sites of expression. In skin, relatively high values were found for *crp4* and *crp5*. After SVCV infection, all *crp*s were mostly upregulated, except for *crp1,* which was mainly downregulated. Predominantly, the highest increases occurred at 5 dpi and in the liver. Significant upregulations were also found in skin for *crp5*–*7*. Additional experiments were completed to further characterize the reactivity of *crp*s to SVCV in skin; they included the use of *rag1* mutants to additionally explore the response of *crps* levels at a both the mucosal and SVCV entry sites in the absence of adaptive immunity. In comparison to *rag1^+/+^* control zebrafish, *rag1^−/−^* mutants showed elevated basal levels of most *crp*s but unaltered *crp6* and *7* levels and the downregulated *crp2*. Furthermore, SVCV infection increased just moderately the expression of most *crp* genes, except for *crp2* and *crp6,* which remained unaltered. The analysis of the transcript expression of all *mx* isoforms on these samples indicates a generalized elevation in skin of the basal status of the innate immune system in fish without adaptive immunity. Altogether, the results obtained confirm the reactivity of *crp*s to viral infections and suggest the skin, and by extension the skin mucus, as a promising sampling source for biomarker testing purposes.

## Data Availability

The data presented in this study are available on request from the corresponding author.

## Supplementary Materials

The following are available online at https://www.mdpi.com/2079-7737/10/2/78/s1, Table S1: qPCR primer sequences for zebrafish genes and SVCV *n*, Figure S1: Expression modulation of *mxa*-*g* in skin from rag mutant zebrafish in response to SVCV infection.
