# Supplementary material for: Modulation of the Tissue Expression Pattern of Zebrafish CRP-Like Molecules Suggests a Relevant Antiviral Role in Fish Skin
Source: Biology (Basel). 2021 Jan 22;10(2):78. doi: 10.3390/biology10020078 (PMC7912335; doi:10.3390/biology10020078)
Supplement: Supplementary file 1 [file biology-10-00078-s001.pdf]

Modulation of the Tissue Expression Pattern of Zebrafish CRP-like Molecules Suggests a Relevant Antiviral Role in Fish Skin

Melissa Bello-Perez [1], Mikolaj Adamek [2], Julio Coll [3], Antonio Figueras [4], Beatriz Novoa [4] and Alberto Falco [1,*]

[1] Institute of Research, Development, and Innovation in Healthcare Biotechnology in Elche (IDiBE), Miguel Hernández University (UMH), 03202 Elche, Spain; melissa.bello@alu.umh.es (M.B.-P.); alber.falco@umh.es (A.F.2)

[2] Fish Disease Research Unit, Institute for Parasitology, University of Veterinary Medicine, 30559 Hannover, Germany; mikolaj.adamek@tiho-hannover.de (M.A.)

[3] Department of Biotechnology, National Agricultural and Food Research and Technology Institute (INIA), 28040 Madrid, Spain; juliocoll@inia.es (J.C.)

[4] Institute of Marine Research, Consejo Superior de Investigaciones Científicas-CSIC, 36208 Vigo, Spain; antoniofigueras@iim.csic.es (A.F.1); beatriznovoa@iim.csic.es (B.N.)

* Correspondence: alber.falco@umh.es; Tel.: +34-966-658-744

Supplementary Materials

Summary:

Table S1: qPCR primer sequences for zebrafish genes and SVCV *n*

Figure S1: Expression modulation of *mxa-g* in skin from *rag* mutant zebrafish in response to SVCV infection.

Table S1. qPCR primer sequences for zebrafish genes and SVCV *n*.

Gene	Sequence (5′-3′)	Accession Number	Reference
ef1a	Fw: CCACGTCGACTCCGGAAA<br>Rv: CGATTCCACCGCATTTGTAGA	AY422992.1	[23]
crp1	Fw: GCCTACCGAACACCCGACT<br>Rv: GCAGGTTGAAAAACGCC	XM_693995	[25]
crp2	Fw: TCTTCTGTTCCCGACTG<br>Rv: GGATGATCTCCCTTTTGG	XM_005162877	[25]
crp3	Fw: ATCCCAGTTATGTTCAAATCG<br>Rv: AGCAGCTCCAACCTG	XM_017353794	[25]
crp4	Fw: GAAAAGTGCTTCTGTTTACAG<br>Rv: CGAACAAGATGACTTCCC	KJ184331	[25]
crp5	Fw: GTGCTTCAGTTCAAGACG<br>Rv: GATGACCTCCCTATCGAG	KC416628	[25]
crp6	Fw: GAACTCAATGTGTGGAGAC<br>Rv: AGATAGAAACTTGCTGGATTG	XM_009297633	[25]
crp7	Fw: CCAAACTGCTACCAGC<br>Rv: AGAATGACTTCCCGCC	KJ184335	[25]
mxa	Fw: GAGACAATCAACCTGGTC<br>Rv: AGTCCTTTCGCCATCA	NM_182942.4	This study
mxb	Fw: GATGTTCATTACCAAGCAG<br>Rv: TCCTTTCGCCCTCG	AJ544824.2	This study
mxc	Fw: AGATGGCATCCACAGTC<br>Rv: TATAGCCCTTCTCTAGGC	NM_001007284.2	This study
mxd	Fw: ATGTTGGAGATCAGATCAAAC<br>Rv: GTCTACGTTTTGTGCCATTC	AJ544826.1	This study
mxe	Fw: CAGGTCACTTCTTGAAGAC<br>Rv: AGTCCTCTAAGATCAGCAG	NM_182867.1	This study
mxf	Fw: TTGGAGATCAGATCAAATCC<br>Rv: CCTTCTGGGTCAACTTG	XM_684467.3	This study
mxg	Fw: GCCATTGTCCAGAACAAG<br>Rv: GTCCAGAGAAATGTCATCATC	NM_001122971.1	This study
**SVCV *n***	Fw: GCATTATGCCGCTCCAAGAG<br>Rv: AGCTTGCATTTGAGATCGA	U18101	[23]

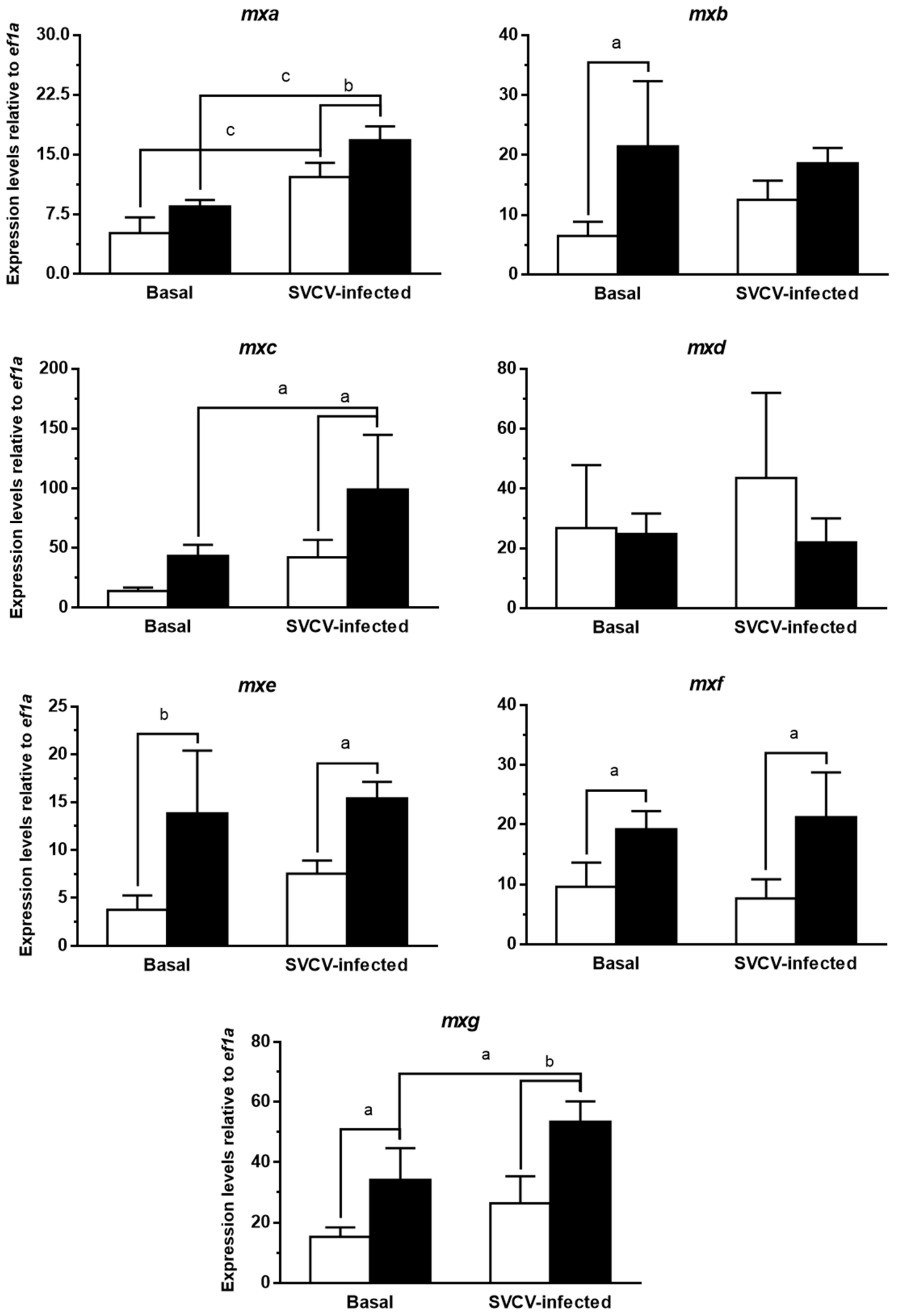

Figure S1. Expression modulation of *mxa-g* in skin from *rag* mutant zebrafish in response to SVCV infection. The transcription levels of *mxa-g* were quantified by RT-qPCR in the skin of *rag*$^{+/+}$ and *rag*$^{-/-}$ mutant zebrafish at 2 dpi with SVCV. *ef1a* mRNA was used as endogenous control to normalize data, which are represented as the mean relative expression level (× 10^3 for *crps*) ± SD of four different individuals. Significant differences were determined by two-way ANOVAs and Sidak's multiple comparison test. Statistical differences between the experimental groups are represented by keys together with 'a', 'b' and 'c' letters on top. a, $P \leq 0.05$; b, $P \leq 0.01$; c, $P \leq 0.001$.
